# Identifying adeno‐associated virus (AAV) vectors that efficiently target high grade glioma cells, for *in vitro* monitoring of temporal cell responses

**DOI:** 10.1002/2211-5463.13894

**Published:** 2024-09-10

**Authors:** Farhana A. Sarker, Yuyan Chen, Adrian Westhaus, Leszek Lisowski, Geraldine M. O'Neill

**Affiliations:** ^1^ Children's Hospital Clinical School, Faculty of Medicine and Health University of Sydney Australia; ^2^ Children's Cancer Research Unit The Children's Hospital at Westmead Sydney Australia; ^3^ Translational Vectorology Research Unit, Faculty of Medicine and Health, Children's Medical Research Institute The University of Sydney Westmead Australia; ^4^ Australian Genome Therapeutics Centre Children's Medical Research Institute and Sydney Children's Hospitals Network Westmead Australia; ^5^ Laboratory of Molecular Oncology and Innovative Therapies Military Institute of Medicine Warsaw Poland

**Keywords:** AAV, barcoded, glioma, YAP/TAZ

## Abstract

To improve the translation of preclinical cancer research data to successful clinical effect, there is an increasing focus on the use of primary patient‐derived cancer cells with limited growth in culture to reduce genetic and phenotype drift. However, these primary lines are less amenable to standardly used methods of exogenous DNA introduction. Adeno‐associated viral (AAV) vectors display tropism for a wide range of human tissues, avidly infect primary cells and have a good safety profile. In the present study, we therefore used a next‐generation sequencing (NGS) barcoded AAV screening method to assess transduction capability of a panel of 36 AAVs in primary cell lines representing high‐grade glioma (HGG) brain tumours including glioblastoma (GBM) and diffuse intrinsic pontine glioma (DIPG)/diffuse midline glioma (DMG). As proof of principle, we created a reporter construct to analyse activity of the transcriptional co‐activators yes‐associated protein (YAP) and transcriptional co‐activator with PDZ‐binding motif (TAZ). Transcriptional activation was monitored by promoter‐driven expression of the Timer fluorescent tag, a protein that fluoresces green immediately after transcription and transitions to red fluorescence over time. As expected, attempts to express the reporter in primary HGG cells from plasmid expression vectors were unsuccessful. Using the top candidate from the AAV screen, we demonstrate successful AAV‐mediated transduction of HGG cells with the YAP/TAZ dynamic activity reporter. In summary, the NGS‐screening approach facilitated screening of many potential AAVs, identifying vectors that can be used to study the biology of primary HGG cells.

## Graphical abstract

Primary patient‐derived cancer cells better emulate human physiology, improving research outcomes. However, delivery of recombinant DNA is a challenge using standard transduction methods. We report a high‐throughput screen to identify an optimal adeno‐associated virus (AAV) vector. We then provide proof‐of‐principle evidence with AAV transduction of primary high‐grade glioma brain cancer cells encoding a novel reporter for measuring YAP/TAZ dynamic signalling.

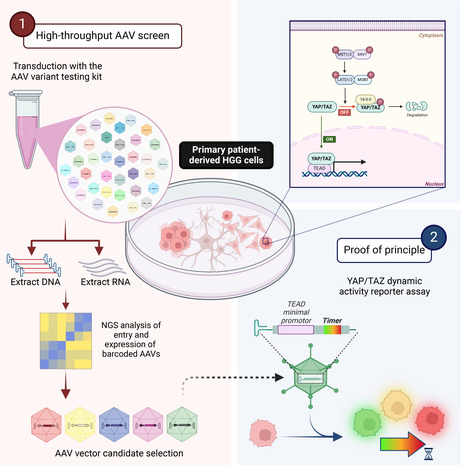

**Abbreviations**
AAV: Adeno‐associated virus
DIPG: Diffuse Intrinsic Pontine Glioma
DMG: Diffuse Midline Glioma
eGFP: enhanced green fluorescent protein
GBM: Glioblastoma
HGG: High grade glioma
HIP: Hippo‐YAP signalling incompetent promoter
HOP: Hippo‐YAP signalling optimal promoter
ITR: Inverted Terminal Repeat
NGS: Next Generation Sequencing
TAZ: Transcriptional coactivator with PDZ‐binding motif
TEAD: Transcriptional Enhanced Associated Domain
VCN: Vector genome copy number
YAP: Yes‐Associated Protein

High‐grade glioma (HGG) are deadly brain tumours that arise from the supporting glial cells of the central nervous system. Presently, there is very little chance of survival past 2 years from diagnosis. A key component to the high mortality of HGG is a rapid and diffuse pattern of growth, where individual and small clusters of tumour cells infiltrate into the healthy brain, destroying vital tissue and evading primary treatments [1]. Paediatric HGG including diffuse intrinsic pontine glioma (DIPG: now reclassified as diffuse midline glioma DMG) were previously grouped with their adult counterparts due to similar clinical and histopathological presentation. However, it has become apparent that paediatric tumours are molecularly distinct and may represent a subset of developmentally derived cancers [2]. Many preclinical studies in HGG have been shown to poorly predict subsequent activity in phase I trials and further to over‐estimate anti‐tumour activity [3]. It is increasingly appreciated that the standard cell lines that have been used in preclinical testing—which have undergone innumerable passages and are cultured in the presence of serum—are phenotypically and genotypically distant from the original source tumour [4]. There is thus a greater focus on low‐passage, serum‐free cell lines cultured directly from patient tumour tissue [5, 6].

We recently demonstrated that the mechanotransductive transcriptional co‐activators yes‐associated protein (YAP) and transcriptional co‐activator with PDZ‐binding motif (TAZ) display rigidity‐dependent nuclear translocation in HGG lines [7]. Translocation of these molecules into the nucleus and subsequent interaction with transcriptional enhanced associated domain (TEAD) transcription factors results in activation of canonical targets [8]. As this is a dynamic process, and since invading cancer cells are likely to be exposed to different tissue stiffness when transmigrating diverse tissues, we were motivated to establish a method for transducing primary HGG cells with a YAP/TAZ reporter that would allow monitoring of this dynamic process.

Primary HGG cell lines present an experimental challenge, as primary cells are resistant to transfection using plasmid‐based methods. Adeno‐associated viral (AAV) vectors are small, non‐enveloped viruses consisting of a total genome size of 4.7 kb, and the dimeric self‐complementary vectors used in this study have a packaging capacity of up to 2.4 kb [9]. AAV variants are encapsulated within different icosahedral capsids that confer cell and tissue tropism [10]. We sought to identify AAV variants with the highest tropism for patient‐derived HGG cells from a broad panel of 36 individually barcoded AAV capsid variants using a high‐throughput, NGS‐based detection method [11]. This identified AAV KP1 and AAV KP3 as efficient gene transfer vectors in all HGG cells tested. As proof of principle, we demonstrate transduction of HGG cells with an AAV KP1::YAP/TAZ activity reporter, carrying a modified GFP (Timer) that has been engineered to colour shift from green to red over time.

## Materials and methods

### Cell culture

Primary patient‐derived HGG cell lines [Glioblastoma (GBM): JK2 and WK1 [5], kindly provided by Bryan Day and Brett Stringer, Queensland Institute of Medical Research; Diffuse Intrinsic Pontine Glioma (DIPG)/Diffuse Midline Glioma (DMG): SU‐DIPG‐6 and SU‐DIPG‐24 [6], kindly provided by Michelle Monje, Stanford University] were maintained in serum‐free defined media for a maximum of 35 passages. Cell line identities were independently verified by STR profiling (Cell Bank Australia). GBM lines were grown as adherent cultures on 1% (v/v) Matrigel‐coated tissue culture plates in KnockOut™ DMEM/F‐12 supplemented with recombinant Human EGF (20 ng·mL^−1^), recombinant human bFGF (10 ng·mL^−1^), glutamine (20 mm·mL^−1^), penicillin/streptomycin (100 U·mL^−1^), StemPro Neural Supplement (20 ng·mL^−1^) and Heparin (20 ng·mL^−1^; Sigma‐Aldrich, Darmstadt, Germany). All media and supplements with the exception of Heparin were purchased from Thermo Fisher Scientific, USA. DIPG/DMG lines were cultured in Tumour Stem Medium (TSM) made from 1 : 1 parts of Neurobasal A medium (Thermo Fisher Scientific, Waltham, MA, USA) and DMEM F‐12 (Thermo Fisher Scientific), with the addition of HEPES (10 mm; Thermo Fisher Scientific), Sodium Pyruvate (1.0 mm; Thermo Fisher Scientific), Non‐essential Amino Acids Solution (1×; Thermo Fisher Scientific), GlutaMAX (100×; Thermo Fisher Scientific) and Antibiotic‐antimycotic (100×; Thermo Fisher Scientific), freshly supplemented with B27‐without vitamin A supplement (Thermo Fisher Scientific), human recombinant EGF (20 ng·mL^−1^; Shenandoah Biotechnology, USA), human recombinant bFGF (20 ng·mL^−1^; Shenandoah Biotechnology, Warminster, PA, USA), human recombinant PDGF‐AA (10 ng·mL^−1^; Shenandoah Biotechnology), human recombinant PGDF‐BB (10 ng·mL^−1^; Shenandoah Biotechnology) and Heparin (2 μg·mL^−1^; StemCell Technologies, Vancouver, BC, Canada).

### AAV testing kit

The AAV variant testing kit containing a mix of 36 iodixanol‐purified AAV capsid variants was provided by the Vectorology Facility [Children's Medical Research Institute (CMRI), Westmead, Australia]. The variants contained within the kit constitute 30 that were previously summarised [11], along with six additional variants AAV KP1, AAV KP2, AAV KP3, AAV NP84, AAV SYDO1 and AAV NP6, details of which have been previously summarised [12]. Each AAV variant contains a unique, random six‐nucleotide barcode (“N6BC”) positioned between an enhanced green fluorescent protein (eGFP) reporter gene and a Woodchuck‐Hepatitis virus post‐translational regulatory element (WPRE). This positioning enables the barcode to relay, via NGS, whether the vector has been successfully delivered into the target cell and if it can drive transgene expression, and for cells to be treated with a mixture of all variants at once. AAV capsids were quantified by real‐time quantitative PCR (RT‐qPCR) with iTaqTM Universal SYBRR green Supermix (Bio‐Rad, Hercules, CA, USA) eGFP primers (forward primer: 5′‐TCAAGATCCGCCACAACATC‐3′; reverse primer: 5′‐TTCTCGTTGGGGTCTTTGCT‐3′) and compared to a standard curve generated from serial dilutions of a known linearised plasmid. AAV variants were first digested with 0.1 unit DNase I (New England Biolabs, Ipswich, MA, USA) in 0.1% (v/v) Pluronic F68 (Thermo Fisher Scientific) at 37 °C for 30 min, followed by incubation with 0.2 μg·μL^−1^ Proteinase K in 0.1% (v/v) Pluronic F68 at 55 °C for 30 min, and denaturing at 95 °C for 15 min. Samples were then mixed with 1% (v/v) Tween 20 for neutralisation, which were further diluted into 1 : 1000, 1 : 10 000 and 1 : 100 000 in Ambion RT‐PCR Grade Water (Thermo Fisher Scientific) for RT‐qPCR quantification. Each variant was then diluted [dilution buffer: 10% v/v Pluronic Acid F68, 1× phosphate‐buffered saline (PBS), 1 m NaCl] to the lowest capsid titre prior to mixing all variants at a 1 : 1 molar ratio. Final variant proportions were confirmed with RT‐qPCR again and independently by NGS (GENEWIZ Genomics, Suzhou, China) using an Illumina HiSeq/MiSeq instrument. Analysis was conducted as previously described [11] and detailed below.

### AAV transduction and assessment of transduction efficiency

Cells were seeded at 2 × 10^5^ per well of a 24‐well culture dish. After overnight incubation, cells were transduced with the AAV mix using a range of dilutions. GFP expression was monitored using an Olympus IX18 inverted microscope. WK1, JK2 and DIPG6 cell lines were incubated with the AAV mix overnight, whilst DIPG24 was incubated for 6 h as following longer incubation periods, cells were detached and cellular debris evident. The AAV mixture was subsequently removed from cells, followed by two washes in 1× PBS solution. Cells were harvested 48 h after transduction via enzymatic detachment with Accutase (Sigma‐Aldrich) and pelleted at 300 ***g*** for 5 min in an Allegra x‐12 centrifuge. DNA and RNA were then extracted from cell pellets using QIAGEN AllPrep DNA/RNA Mini Kit (QIAGEN, Hilden, Germany). Concentration and purity were assessed with the NanoDrop™2000 spectrophotometer and DNA integrity confirmed by agarose gel electrophoresis. Transduced AAV vector copy number was quantified by digital droplet PCR (ddPCR) analysis of eGFP (forward primer: 5′‐TCAAGATCCGCCACAACATC‐3′; reverse primer: 5′‐TTCTCGTTGGGGTCTTTGCT‐3′) normalised to human albumin (forward primer: 5′‐ TGCTGTCATCTCTTGTGGGCTG‐3′; reverse primer: 5′‐ AACTCATGGGAGCTGCTGGTTC‐3) using QX200™ ddPCR EvaGreen Supermix, QX200™ Droplet Generator and Droplet Reader, followed by analysis using QuantaSoft™ software (ver. 1.7.4) (all from Bio‐Rad). The efficacy of entry efficiency and expression of each AAV variant was assessed through NGS of transduced HGG gDNA and mRNA/cDNA, respectively. Extracted total RNA (~ 1 μg) was incubated with 2 units of TURBO DNase (Thermo Fisher Scientific) for 1 h at 37 °C followed by inactivation with the TURBO DNase inactivation reagent at room temperature. RNA was then treated with the SuperScript IV First‐Strand Synthesis System (Thermo Fisher Scientific) and 2 μm WRPE‐binding primer (5′‐GGATTTATACAAGGAGGAGAAAATGAAAG‐3′) following manufacturer's instructions.

Next, primers were designed to generate 155 bp amplicons containing configurations of the N6BC barcodes that were associated with transduced AAV variants. Primers N6BC‐NGS_F (forward: 5′‐NNNNNGCTGGAGTTCGTGACCGCCG‐3′; where “NNNNN” represents different barcodes) and N6BC‐NGS_R (reverse: 5′‐CAACATAGTTAAGAATACCAGTCAATCTTTCAC‐3′) [11] were incubated with 50 ng of extracted gDNA/5 μL cDNA and Q5 High‐Fidelity DNA Polymerase (New England Biolabs, Ipswich, MA, USA). Samples were then cycled with the following PCR program: denaturation at 98 °C for 2 min, 42 amplification cycles at 98 °C for 10 s, 62 °C for 10 s and 72 °C for 10 s, followed by a final extension at 72 °C for 10 min. The PCR products were separated on a 2% (w/v) Tris‐acetate‐EDTA (TAE) gel. Target bands were excised extracted using a FavorPrep™ Gel Purification Mini Kit (Favorgen, Pingtung County, Taiwan). PCR products from all cell lines were pooled into one sample comprising 250 ng DNA each and sent to GENEWIZ Genomics. Analysis of paired reads was conducted offsite with BBMerge Paired Read Merger (v.37.64) and Geneious Workflow (Geneious, v11.1.4). NGS results were deconvoluted with a custom Python‐based N6BC barcode identifier and counter script [11]. Read counts for each cell line and each variant were multiplied by a variant‐specific “normalisation coefficient” [% of NGS reads (ideal)/% of NGS reads (measured)]. The percentage of each AAV variant present within each sample was then determined by dividing each variant normalised read by the sum of all variant normalised reads. This was computed for all cell lines using Excel and data plotted with gra (v9.0.1; Boston, MA, USA).

### Flow cytometric analyses

Cells were seeded in 24‐well dishes (WK1: 200 000 and DIPG24: 150 000) and transduced with 10 000 and 50 000 MOT of each AAV in duplicate wells, as indicated. At 48 h following transduction, cells were harvested and cell pellets were resuspended with chilled FACs buffer (137.4 mm NaCl, 2.7 mm KCl, 10.1 mm Na_2_PO_4_, 1.8 mm KH_2_PO_4_, 0.1% (w/v) BSA) and passed through the 35 μm cell strainer‐cap of a 5 mL FACs tube (Corning™, Corning, NY, USA). Samples were then immediately processed on a FACs Canto II cytometer (BD Biosciences, Franklin Lakes, NJ, USA), collecting 10 000 events for each. For all samples, a forward scatter height (FSC‐H) and area (FSC‐A) gating strategy was used to exclude any doublet cells. The subpopulation of single cells was then gated against the B530_30E‐A fluorochrome detector to determine the percentage of eGFP‐positive cells, with non‐transduced cells serving as the negative control. Subsequent analysis was conducted with FlowJo™v10.8 software (FlowJo LLC, Ashland, OR, USA).

### HIP/HOP‐timer expression vector construction

Plasmids encoding luciferase reporters downstream of multiple copies of promoter sequences containing TEAD binding sites [13] were sourced from Addgene (Watertown, MA, USA) [Hippo‐YAP signalling optimal promoter (HOP)‐flash #83467; negative control Hippo‐YAP signalling incompetent promoter (HIP)‐flash #83466]. Exchange of luciferase for the Timer reporter (a mutant form of the red‐fluorescent protein DS‐RED that changes colour over time [14], purchased from TaKaRa Bio, Shiga, Japan) in the HIP‐ and HOP‐Flash plasmids was conducted by GenScript^®^ (Piscataway, NJ, USA). Briefly, luciferase sequences were exchanged for the Timer open reading frame, generating constructs in which the HIP (negative control) or HOP (positive) promoter sequences were located upstream of Timer, with the new plasmids labelled HIP‐ and HOP‐Timer. To generate AAV vectors encoding the HIP‐/HOP‐Timer constructs, a transfer plasmid containing the transgenes and structural AAV Inverted Terminal Repeat (ITR) regions (scAAV‐H1‐RSV‐eGFP) was engineered by exchanging the promotor and reporter encoding regions for an existing RSV‐eGFP between the ITR sites of an existing self‐complementary (sc) AAV expression cassette backbone. scAAV‐H1‐RSV‐eGFP was digested with restriction enzymes AscI and XhoI, and the backbone (~ 3.2 kb) was assembled with two PCR fragments (one containing HIP‐ or HOP‐Timer amplified from HIP‐ or HOP‐Timer constructs as above (~ 1.2 kb), the other containing “Stuffer DNA” amplified from an existing plasmid (T3 Stuffer_LSP_Cerulean HCP consisting of ~ 350 bp of unrelated DNA sequence)) using NEBuilderR HiFi DNA Assembly Cloning Kit (New England Biolabs, Ipswitch, MA, USA). Primers were designed to contain overlap sequences that adjoined the ITR regions of the scAAV expression cassette to the HIP/HOP‐pTimer transgene and stuffer DNA (Fragment 1 F: 5′‐TCCCAGATCTGATATCGGCGCGCCGGTACCGAGCTCGGAT‐3′ and Fragment 1 R: 5′‐GTCGACGTCGACTCCGGC‐3′; Fragment 2 F: 5′‐GTCGACGTCGACGGATCCTTATCGA‐3′ and Fragment 2 R: 5′‐TATCGTCGAGGCCGCCTCGAGCGACGTCTGTATAGGGATG‐3′). Prior to packaging, quality control of the assembled scAAV::HIP‐/HOP‐Timer transfer plasmids was conducted, via diagnostic restriction enzyme digestion to ensure ITR region integrity (AdhI, MscI, XmaI cleavage), Sanger sequencing (Australian Genome Research Facility) and ddPCR as described above to confirm transfer plasmid concentrations. AAV packaging of the new AAV::HIP‐/HOP‐Timer reporter constructs was conducted by Vectorology Services at the Translational Vectorology Group using Iodixanol Gradient Purification. The final AAV products were also quantified with ddPCR as described above.

## Results

### Screening AAV vectors for HGG cell transduction

**Fig. 1:**
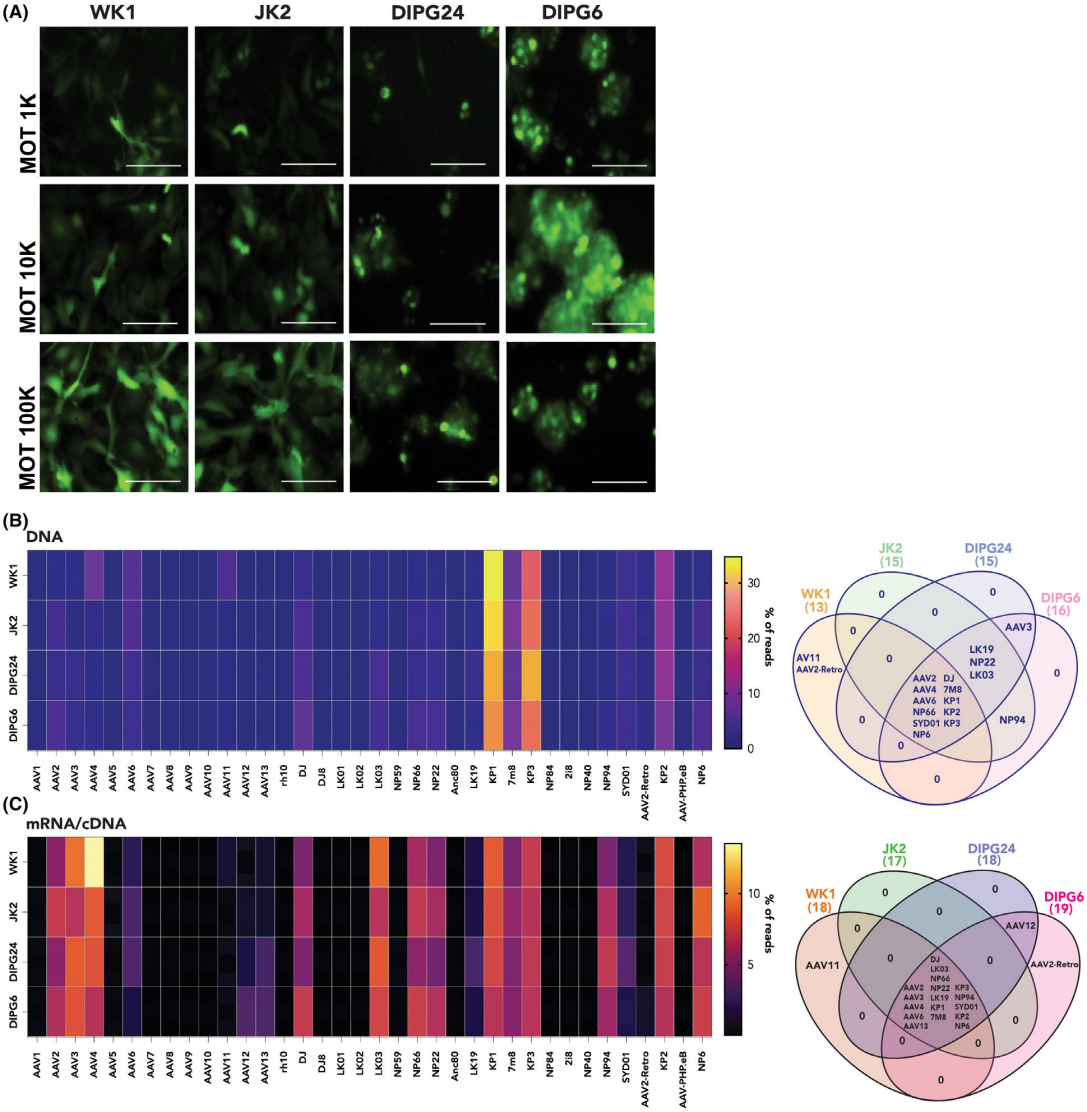
Screen identifies AAV variants with tropism for primary patient‐derived HGG cells. (A) Representative fluorescence images of GFP‐positive cells in primary, patient‐derived HGG cell lines, WK1, JK2, DIPG24 and DIPG6 transduced with 1 : 1 molar mixture of 36 individual AAV variants with the indicated MOTs. Scale bars = 100 μm. (B) NGS‐based quantification of N_6_BC barcodes of individual AAV variants in DNA extracted from transduced HGG cell lines to quantify transduction efficiency of each variant into cells. Values represent percentage of total NGS‐DNA reads for each variant within each cell line. Look‐up table represents low (blue) to high (yellow) percentage. Adjacent Venn diagram shows distribution of best‐performing AAV variants (> 1% of total NGS‐DNA reads). (C) Results of NGS‐based quantification of barcoded variants from vector genome‐transcribed mRNA/cDNA to determine functional transduction efficacy. Values are percentage of total variant reads for each cell line. Look‐up table scales low (black) to high (yellow) percentage. Venn diagram shows distribution of highly expressed AAV variants (> 5% of total NGS‐RNA reads).

To identify AAV vectors suitable for transducing HGG cells, a panel of 36 individually barcoded AAV capsid variants were screened utilising a high‐throughput, NGS‐based detection approach [11]. HGG cells were transduced with a 1 : 1 molar ratio mixture of all AAV variants simultaneously. Primary patient‐derived HGG cell lines JK2, WK1, DIPG6 and DIPG24 were transduced with the AAV Testing Kit. The cells were transduced at total MOT of 1000, 10 000 and 100 000 vector genomes per cell (vg per cell), equivalent to individual MOT of ~ 28, 278 and 2778 per AAV variant. Notably, during culture, the DIPG lines characteristically grow as loosely attached chains that pile up on each other (DIPG24), or as neurospheres (DIPG6) [15], hence the out‐of‐focus appearance of these cultures (Fig. 1A). Both DIPG lines exhibited cell clumping at the highest dose and due to apparent toxicities indicated by cell rounding and debris, DIPG24 cells were only transduced for 6 h. Transduction efficiency was monitored by assessment of GFP‐positive cells (Fig. 1A) and independently quantified by ddPCR‐mediated detection of GFP coding sequence in genomic DNA extracts, normalised to human albumin. Quantification of the average vector genome copy number per diploid genome (VCN·dg^−1^) confirmed transduction of the AAV mix into each of the cell lines investigated (VCN·dg^−1^: WK1 100 000 vg per cell = 1423; JK2 100 000 vg per cell = 134; DIPG6 10 000 vg per cell = 98; DIPG24 10 000 vg per cell = 64). Lower transduction efficiencies in the DIPG24 likely reflect the shorter transduction incubation time.

Next, to elucidate individual AAV variant uptake and expression, respectively, within each cell line, DNA and RNA was extracted, and barcoded variants were analysed by NGS. Genomic DNA was used for NGS analysis of the unique barcode (BC) present in the AAV expression cassette. Analysis of the NGS results revealed that the AAV capsid variants contributing to the average vector copy number for each of the four HGG lines were remarkably consistent (Fig. 1B, Venn diagram). In each cell line, AAV KP1 contributed the greatest fraction ranging from 25% to 35% of all reads, closely followed by AAV KP3 which contributed 20–28% of all reads (Fig. 1B). Thus, almost 50% of the vector uptake was accounted for by these two variants.

We next utilised NGS of the BC region of the expression cassette to quantify transgene expression at the RNA (cDNA) level (Fig. 1C). This revealed that capsids with the highest transduction efficiency (AAV KP1 and AAV KP3) resulted in transgene expression levels that were comparable to those driven by capsids that contributed only to a small fraction of the total genomic DNA reads. Notably, there was a consistent pattern of gene expression by the different capsids across the four cell lines (Fig. 1C). AAV KP1, AAV KP3, AAV 7m8, AAV DJ and AAV 4 were chosen for further analysis due to their combined viral entry and transgene expression, suggesting that they mediate effective delivery into HGG cells. Notably, AAV 9, which is frequently used to deliver therapeutic cargo to the CNS [16] and is the serotype utilised in market‐approved gene therapy for spinal muscular atrophy (SMA) (Zolgensma™) [17], had low efficiency in HGG cells.

### AAV KP1 and AAV KP3 efficiently transduce HGG cells

**Fig. 2:**
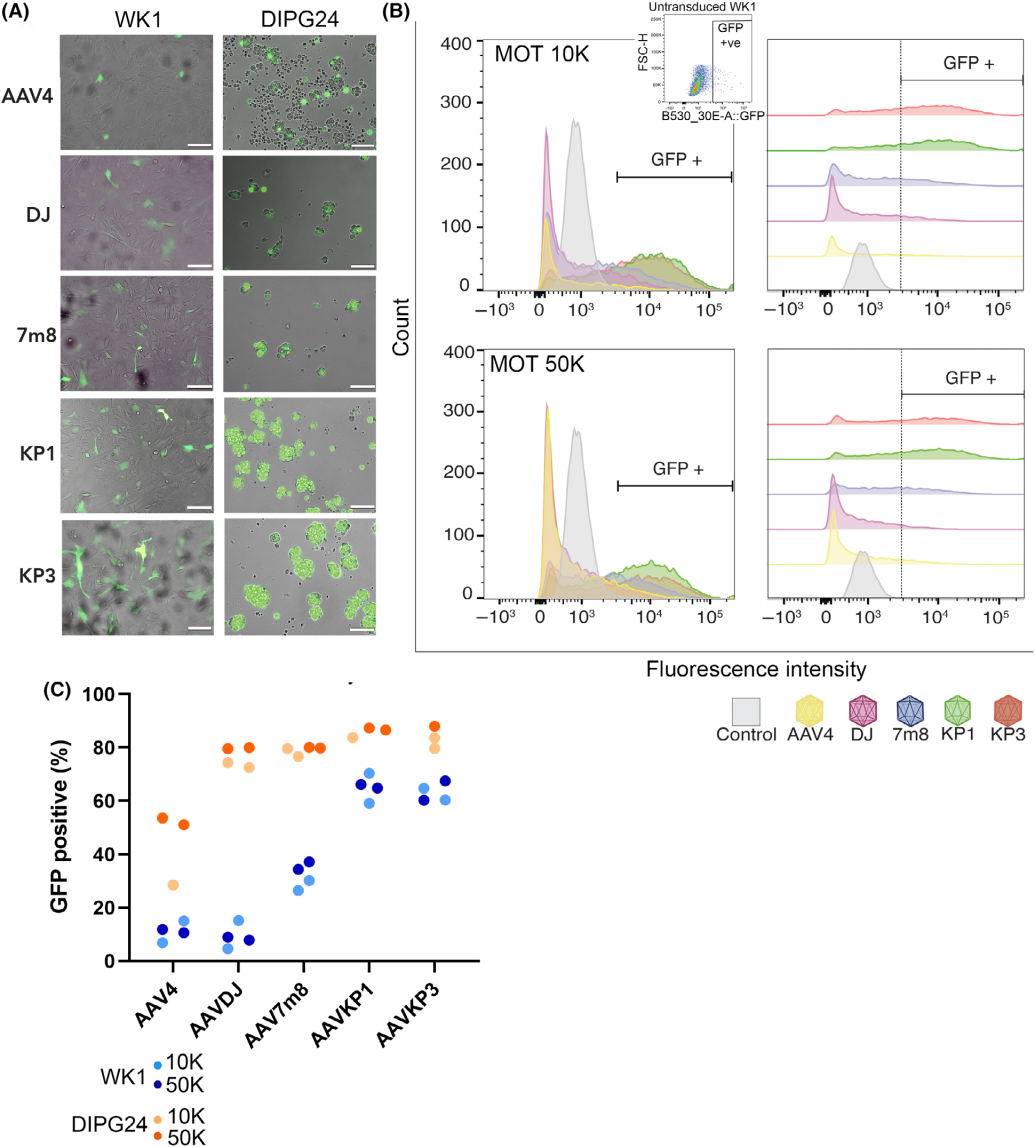
Quantification of transgene expression. (A) Micrographs showing GFP‐positive cells overlayed on bright‐field images of WK1 and DIPG24 cells, transduced as indicated. Images of WK1 cells were acquired 24 h after transduction and DIPG24 6 h after transduction. Scale bars = 200 μm. (B) Representative histograms of GFP‐profiling by flow cytometry, showing fluorescence intensity (*x*‐axis) and cell count (*y*‐axis) of AAV‐transduced WK1 cells (MOT 10K and 50K, as indicated). Colours of the individual histogram plots correspond to the colours for each AAV as per the key at the bottom of the figure. Plots on the right show the same data, with data for each AAV separated in half offset format. Dotted line indicates the delineation between GFP‐positive and GFP‐negative cell populations. Scatter‐plot inset shows example of non‐transduced WK1‐negative control, used to establish gates for GFP positivity. (C) Graph show GFP‐positive cells as a percentage of the total cell population determined by flow cytometry. WK1 (blue) and DIPG24 (orange) cells were transduced with the indicated AAV serotypes at MOTs of 10 000 (light blue and light orange, respectively) and 50 000 (dark blue and dark orange, respectively). Dots represent individual repeats.

Next, we compared GFP expression after transduction with the 5 AAV individual variants identified from the screen as having the best efficiency. To determine account for potential differences between cell lines with different origins, we compared GFP transgene expression between WK1 GBM cells and DIPG24 cells (Fig. 2A). Quantification by flow cytometry (Fig. 2B) revealed that AAV KP1 and AAV KP3 resulted in the greatest number of GFP‐positive WK1 cells (Fig. 2C). GFP expression was low in WK1 cells transduced with AA V4 and AAV DJ vectors and intermediate expression was quantified in WK1 cells transduced with AAV 7m8 (Fig. 2C). This contrasts with DIPG24, where 4/5 AAVs (AAV DJ, AAV 7m8, AAV KP1, AAV KP3) resulted in equivalently high expression, and the 5^th^, AAV 4, resulted in an intermediate level of expression (Fig. 2C). Notably, capsids AAV KP1 and AAV KP3 were consistently high in all functional assessments. AAV KP1 was selected as the variant of choice for further experiments, as more cellular debris was evident following exposure to AAV KP3.

### Transduction with the HIP/HOP‐TIMER

**Fig. 3:**
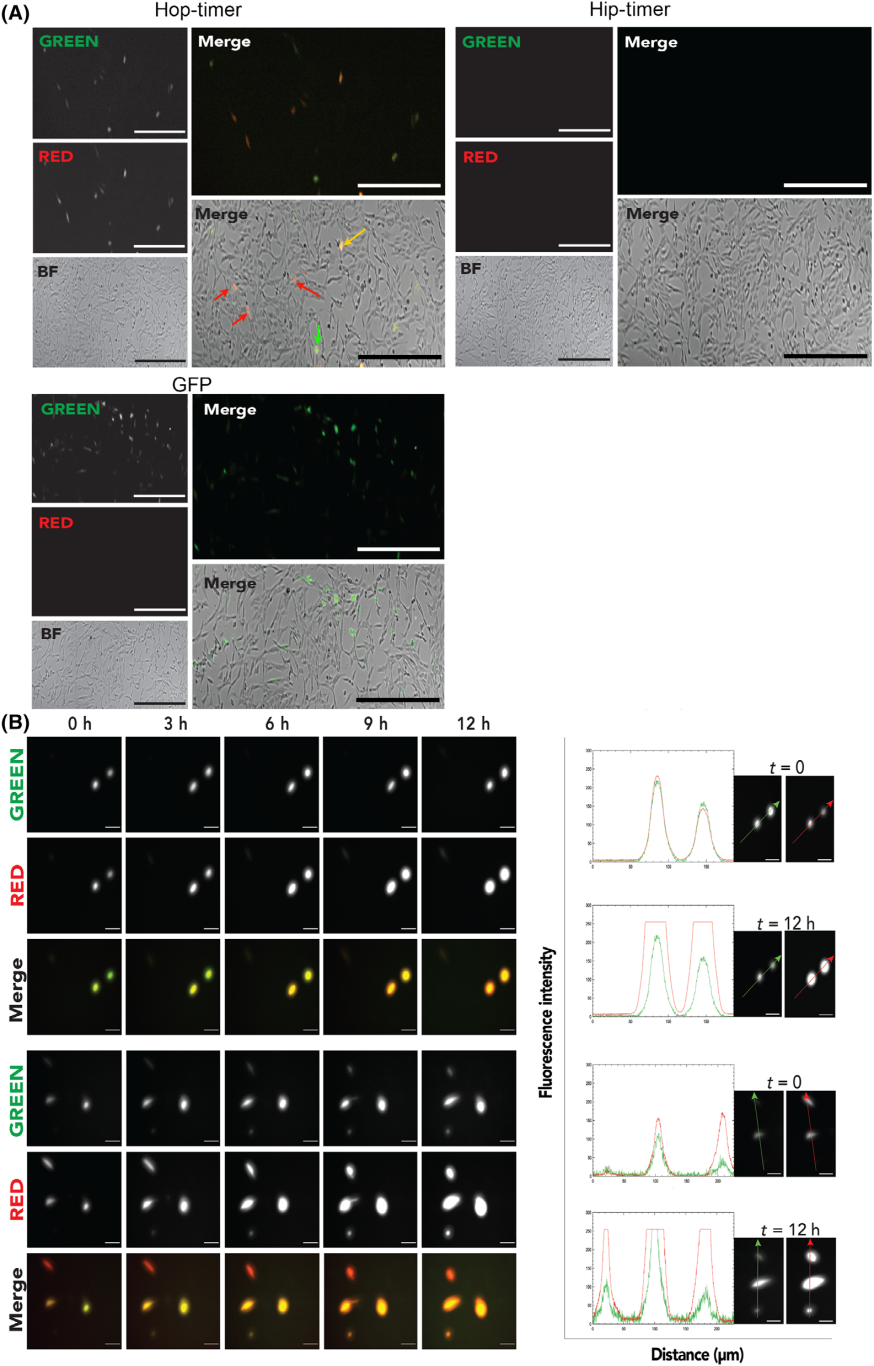
Confirmation of dynamic fluorescent HOP‐Timer expression. (A) Representative fluorescence and bright‐field images of NIH 3T3 fibroblast cells 24 h after transfection with HOP‐/HIP‐Timer plasmid constructs and GFP plasmid control. Arrows in HOP‐Timer transfected cells in the bright‐field and fluorescent image overlay indicate cells in which the Timer reporter protein has recently been synthesised (green arrows to green cells), cells in which the reporter has been expressed for some time (yellow arrows to yellow cells) and in which the reporter has been expressed for the longest period (red arrows to red cells). Scale bars = 100 μm. (B) Representative images of two fields of view of 3T3 fibroblasts transfected with HOP‐Timer. Time‐lapse images of positive cells were captured over a 12‐h period from 24 h post transfection. Line scans to the right depict green and red channel fluorescence intensity of HOP‐Timer expressing cells at the start of imagine (*t* = 0 h) and the end (*t* = 12 h). Arrows in adjacent images depict the position and direction of the line scans. Scale bars = 50 μm.

To generate a dynamic fluorescent reporter for YAP/TAZ activity, the luciferase coding domain of the HIP‐ and HOP‐Flash plasmids was exchanged for the Timer reporter. Functionality of the HIP‐/HOP‐Timer plasmids was assessed by transfection into murine NIH 3T3 fibroblasts and assessed after 24 h (Fig. 3A). Imaging of HOP‐Timer‐transfected cells revealed examples of green‐positive cells representing early reporter translation, yellow cells (mix of red and green) representing longer‐lived reporter molecules and red indicating cells with the longest‐lived Timer expression (Fig. 3A). No fluorescent cells were detected in the HIP‐Timer‐negative control cells. NIH 3T3 cells transfected with a control GFP plasmid displayed greater numbers of positive cells than seen with the HOP‐Timer construct (Fig. 3A). The smaller fraction of the HOP‐Timer‐positive cells likely reflects a combination of transfection efficiency and the percentage of cells with YAP/TAZ activation during the period of the experiment. To confirm the dynamic colour change of the Timer reporter, HOP‐Timer‐transfected cells were analysed across the time‐lapse imaging period. Over 12 h, positive cells transitioned from predominantly green fluorescence to increased red fluorescence (Fig. 3B).

**Fig. 4:**
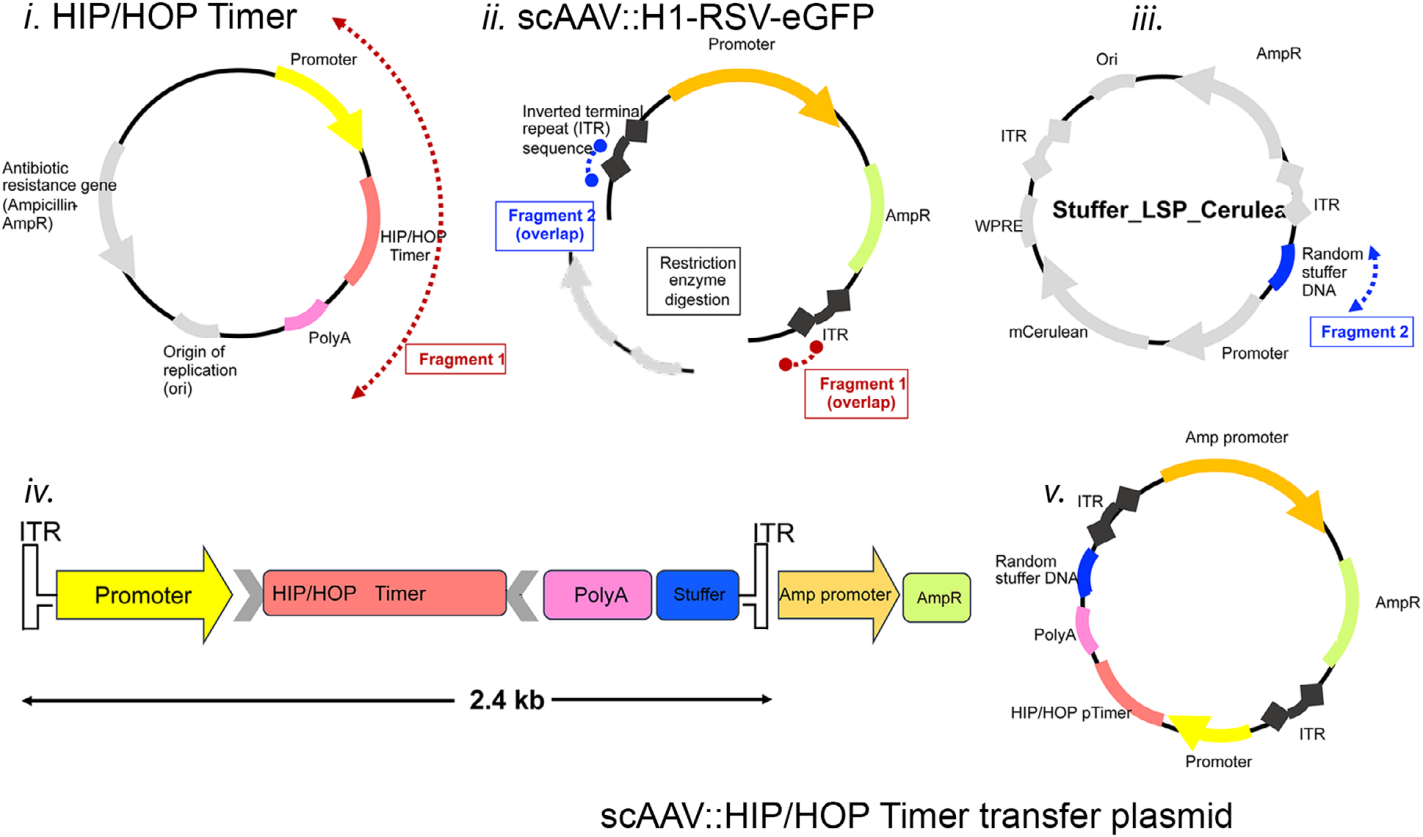
Schematic representation of scAAV::HIP/HOP‐Timer construction. Self‐complementary AAV (scAAV) expression cassette (*ii*: scAAV::H1‐RSV‐eGFP) was used as a backbone for AAV transfer plasmid assembly and prepared through restriction enzyme digestion. Two insert fragments were generated with PCR amplification, Fragment 1 (highlighted in red) was isolated from the HIP/HOP‐Timer plasmid (*i*) and contained the HIP/HOP‐Timer transgene conjugated to an ITR region from the scAAV expression cassette. Fragment 2 (highlighted in blue) contained the second ITR and random DNA base pairs from Stuffer_LSP_Cerulean (*iii*) to buffer remaining space in the final construct. Fragments were assembled into the scAAV expression cassette backbone with the NEBuilder HIFI DNA assembly cloning kit (New England Biolabs, Ipswitch, MA, USA), such that the final scAAV::HIP/HOP‐Timer transfer plasmid (*v*) encoded the HIP/HOP‐Timer transgene flanked between AAV ITR regions (*iv*).

**Fig. 5:**
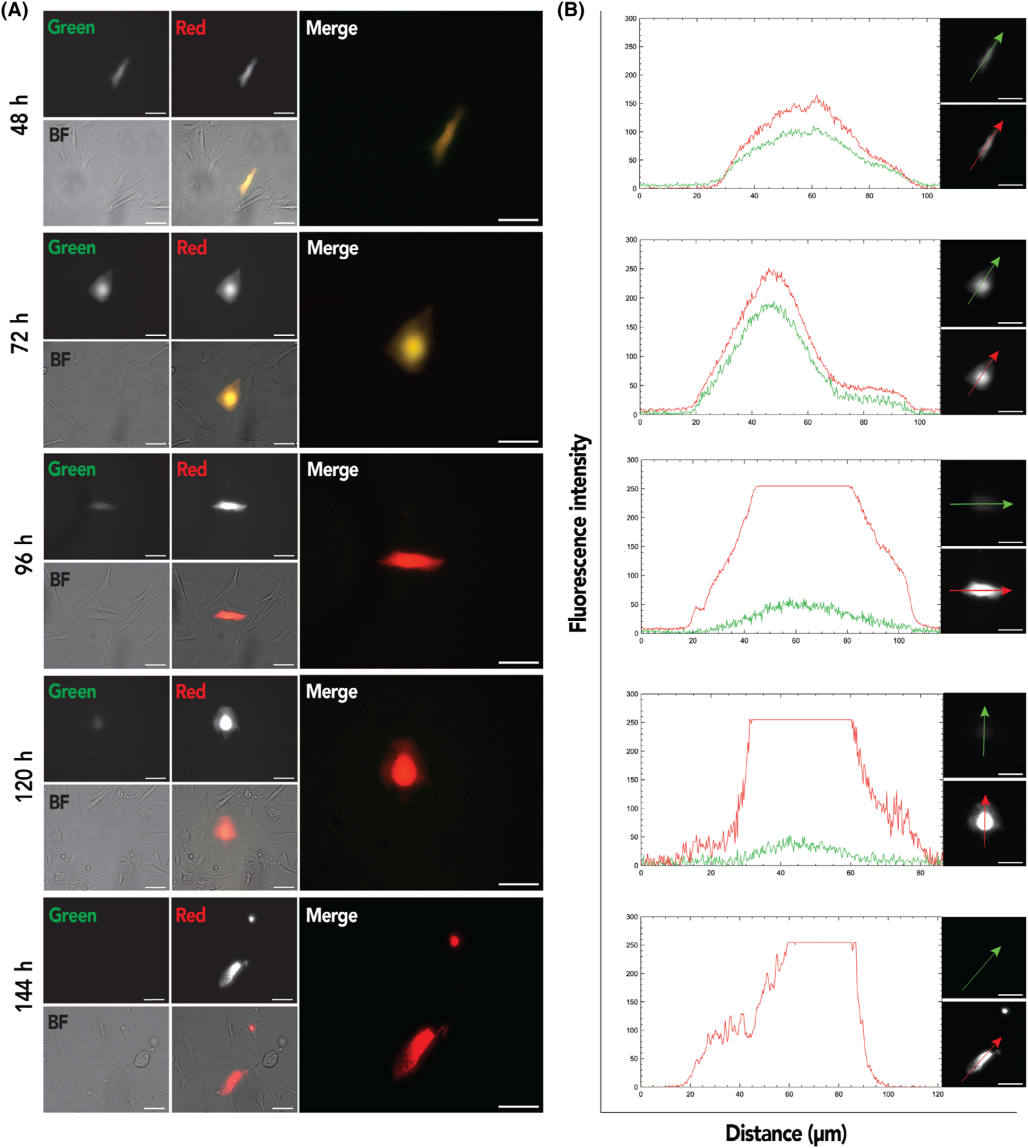
Transduction of WK1 cells with HOP‐Timer delivered by AAVKP1. (A) Representative images of WK1 cells transduced with AAVKP1::HOP‐Timer. Shown are examples of individual cells at the indicated time periods after transduction. Scale bars = 50 μm. (B) Line scans showing green and red fluorescence of HOP‐Timer‐positive cells. Arrows in adjacent images indicate the direction of the line scans. Scale bars = 50 μm.

Attempts to transfect WK1 cells with the HOP‐Timer plasmid using different methods (lipofectamine and nucleofection) were unsuccessful, with no positive cells detected (data not shown). This was not due to lack of YAP/TAZ expression and activity in these cell lines, as we have previously demonstrated YAP/TAZ activity in WK1 cells [7]. This underscores the need for alternative transduction method for these cell lines. Consequently, as proof of principle, HIP/HOP‐Timer transfer plasmids were constructed (Fig. 4) and the HIP‐/HOP‐Timer constructs then packaged into AAV 1KP1 and used to transduce WK1 cells, with an MOT of 50 000. Transduced cultures were observed from 48 h after transduction then every day up to 6 days (Fig. 5A). This revealed a mix of cells exhibiting both green and red fluorescence (appearing yellow in merged images) and cells that were predominantly red (Fig. 5A,B). Cloning of the HOP‐Timer into the AAV KP1 vector therefore provides a method for studying the activity of YAP/TAZ that was not possible with plasmid transfection methods.

## Discussion

In this study, the identification of optimal AAV variant tropism to HGG cell lines was made feasible by multiplexed, barcoded, NGS‐based detection [11]. Cell lines representing GBM and DIPG were assessed for transduction, in case there were significant differences in AAV tropism for these different cell types. Notably, while we found relatively consistent tropism across the 36 variants between the different cancer cell types, subsequent assessment of the top five candidates from the AAV variant screen in individual assessments identified some differences between preference for DIPG versus GBM. Of the 5 top candidates, AAV KP1 and AAV KP3, displayed both high‐level transduction and subsequent functional protein expression in both GBM and DIPG cancer cell lines.

Of the 5 top variants, AAV4 is the only naturally occurring serotype, with the others representing synthetically derived variants. AAV KP1 and AAV KP3 were originally generated from capsid shuffled libraries and selected for high transduction efficiency of primary human islets [18]. The presence of neutralising antibodies due to the prevalence of AAV exposure in the population is a significant complication in the *in vivo* application of AAV vectors [19]. The derivation and application of synthetic variants is one approach taken to overcome this limitation [20]. Neutralisation assays with AAV KP1 and AAV KP3 with pooled human immunoglobin suggested that they required greater concentrations of antibody for efficient neutralisation than AAV LKO3, although this was less than that required for AAV DJ [18]. Thus, in addition to these vectors as promising tools for preclinical analysis in HGG, they may have further application in clinical applications in HGG.

To demonstrate the utility of the AAV vectors for transducing HGG cells, we created a novel YAP/TAZ promoter reporter construct. This novel reporter affords the opportunity to visualise YAP/TAZ activity over time in primary brain cancer cells, for example as cells migrate and invade through environments with different mechanical characteristics. We note that since the first description of the Timer reporter [14], it has become apparent that this molecule has a propensity to aggregate [21]. While this characteristic prevents the use of Timer as a fused tag for analysing protein function in other applications, in the case of the YAP/TAZ Timer, the Timer moiety is unfused and thus is a suitable molecule for analysing promoter activity, even if the molecule undergoes aggregation.

Transduction with AAV KP1 overcame the limitations of transducing primary HGG cells with plasmid expression vectors. Despite successful transduction, there was only a low rate of positive cells, considerably lower than AAV KP1 constructs constitutively expressing GFP control protein. Notably, the cells were analysed under steady‐state conditions, thus YAP/TAZ activity is not synchronised across the population. Therefore, at any time only a percentage of cells are anticipated to display YAP/TAZ mediated activation of the TEAD promoter and hence Timer expression. Nonetheless, the data demonstrate that AAV can successfully transduce difficult‐to‐transfect primary HGG cells, opening the way for future analysis of temporally regulated pathways in living cells.

## Conflict of interest

The authors declare no conflict of interest.

## Author contributions

Conceived the project: LL and GON; experimental design: FAS, YC, AW, LL and GON; undertook experiments: FAS, YC and AW; interpreted the data FAS, YC and AW; drafted the manuscript: FAS and GON; revised and approved the final manuscript: FAS, YC, AW, LL and GON.

## Data Availability

Data are available upon request from the corresponding author.
